# Mental Health and Health-Related Behavioral Patterns Surrounding a Major International Sporting Event: An Observational Study

**DOI:** 10.3390/diseases14090318

**Published:** 2026-08-31

**Authors:** Gustavo A. Hernández-Fuentes, Mario A. Alcalá-Pérez, Uriel Díaz-Llerenas, Marcos E. Guerrero-Verduzco, Nancy A. Reyes-Méndez, Laura A. Larios-Gómez, Dalila G. Virgen-Aguilar, Jessica C. Romero-Michel, Verónica M. Guzmán-Sandoval, Fabian Rojas-Larios, Pedro J. Flores-Moreno, Osval A. Montesinos-López, Marina Delgado-Machuca, Iván Delgado-Enciso

**Affiliations:** 1 Department of Molecular Medicine, School of Medicine, University of Colima, Colima 28040, Colima, Mexico; mguerrero24@ucol.mx (M.E.G.-V.); frojas@ucol.mx (F.R.-L.); 2 State Cancerology Institute of Colima, Health Services of the Mexican Social Security Institute for Welfare (IMSS-BIENESTAR), Colima 28085, Colima, Mexico; dalilavirgen6@gmail.com; 3 Faculty of Chemical Sciences, University of Colima, Coquimatlan 28400, Colima, Mexico; llarios@ucol.mx; 4 Molecular Medicine Laboratory, Academic Unit of Human Medicine and Health Sciences, Autonomous University of Zacatecas, Zacatecas 98160, Zacatecas, Mexico; marioalcalaperez@uaz.edu.mx (M.A.A.-P.); urieldiazllerenas@gmail.com (U.D.-L.); 5 Carrera de Medicina Integral y Salud Comunitaria, Sede Armería, Universidad para el Bienestar Benito Juárez García (UBBJ), Degollado 428, Ejido Armería, Colima 28305, Colima, Mexico; 6 Health Department, El Colegio de la Frontera Sur, San Cristóbal de las Casas 29290, Chiapas, Mexico; nancy.reyes@posgrado.ecosur.mx; 7 Universidad Contemporánea de las Américas, Pino Suárez 20, Centro, Acámbaro 38600, Guanajuato, Mexico; 8 Faculty of Law, University of Colima, Colima 28040, Colima, Mexico; jessica_romero@ucol.mx; 9 School of Psychology, University of Colima, Colima 28040, Colima, Mexico; gus_vero@ucol.mx; 10 Faculty of Education Sciences, Universidad de Colima, Av. Universidad 333, Las Víboras, Colima 28040, Colima, Mexico; pedrojulian_flores@ucol.mx; 11 Faculty of Telematics, University of Colima, Colima 28040, Colima, Mexico; oamontes1@ucol.mx; 12 Department of Epidemiology, Family Medicine Unit No. 19, Mexican Social Security Institute (IMSS), Colima 28000, Colima, Mexico; karla_machuca@ucol.mx; 13 Department of Dietetics and Nutrition, Robert Stempel College of Public Health and Social Work, Florida International University, Miami, FL 33199, USA

**Keywords:** mental health, anxiety-related symptoms, depressive-related symptoms, emotional well-being, alcohol consumption, preventive mental health, young adults, physical activity, multidisciplinary care, FIFA World Cup

## Abstract

Background/Objectives: Acute collective emotional events may coincide with variation across multiple dimensions of mental health and health-related behaviors. This study compared mental health outcomes and recent alcohol consumption between assessments conducted before and after a major international sporting event and explored factors associated with psychological and behavioral outcomes among young adults. Methods: An observational study was conducted among 146 adult students in western Mexico who participated in assessments surrounding the 2026 FIFA World Cup match between Mexico and England. Assessments were conducted approximately 10 h before kick-off and 11 h after completion of the match. Because individual responses could not be linked across assessment periods, comparisons were analyzed as repeated cross-sectional marginal comparisons rather than paired longitudinal analyses. Psychological outcomes were assessed using standardized instruments, and recent alcohol consumption was defined as alcohol use during the eight hours preceding each assessment. Results: Anxiety-related symptom scores were lower at the post-event assessment, whereas depressive-related symptoms remained relatively stable. Emotional well-being was also lower at the post-event assessment, while recent alcohol consumption during the eight hours preceding the assessment was substantially more frequent post-event than pre-event (41.1% vs. 0.7%). The small difference observed in sexual-attitude scores was considered exploratory. Post-event multivariable analyses identified several factors associated with psychological outcomes and recent alcohol consumption; these associations were interpreted as cross-sectional. Conclusions: Psychological and behavioral outcomes exhibited distinct patterns between assessment periods surrounding the sporting event. Given the observational design, inability to link individual responses, and presence of unmeasured co-occurring exposures, these differences should not be interpreted as within-participant changes or as effects attributable specifically to the match.

## 1. Introduction

Major international sporting events extend beyond competition and function as collective social experiences capable of eliciting intense emotional, physiological, and behavioral responses among spectators [1,2]. Soccer is particularly relevant in this regard because identification with a national or club team may generate anticipatory tension, emotional contagion, collective celebration, and abrupt changes in affect as the result of a match becomes known [3,4]. Recent studies using wearable devices have shown that soccer spectators may experience increased heart rate and stress beginning several hours before kick-off, with additional fluctuations during decisive phases of a match. These observations support the concept of “soccer fever” as a dynamic response characterized by marked physiological activation and substantial variability among supporters [5,6].

The psychological response to soccer spectatorship is not necessarily limited to the duration of the match. Anticipation of an uncertain outcome may increase anxiety before the event, whereas resolution of that uncertainty may reduce tension once the match has ended [7]. Nevertheless, the post-match period may also be characterized by emotional exhaustion, fatigue, disrupted routines, reduced sleep, or disappointment; these factors may coincide with differences in anxiety and perceived well-being. Moreover, responses may differ according to sex, previous sports participation, personal identification with soccer, and other social or behavioral characteristics [5,8,9].

Although anxiety, stress, alcohol use, and emotional reactions have individually been investigated in relation to sporting events, considerably less attention has been paid to their simultaneous assessment within the same population and temporal context [10]. Examining these domains concurrently may provide a more comprehensive perspective on psychological and behavioral patterns observed within a tightly defined temporal window surrounding major sporting events [7,11].

Alcohol consumption represents another important component of the social context surrounding soccer. Sporting events are frequently watched in groups, homes, bars, restaurants, and fan zones, where alcohol may be incorporated into celebration, social bonding, and match-related rituals [12,13]. Evidence from different settings indicates that spectators may consume substantial quantities of alcohol while watching sporting events, and analyses conducted during previous FIFA World Cup tournaments have identified increased public interest in alcoholic beverages during the competition [12,13]. From a public health perspective, alcohol consumption during major sporting events is relevant because it may coexist with emotional arousal, impaired judgment, dehydration, sleep disruption, interpersonal violence, traffic injuries, and other acute health risks among spectators and surrounding communities. However, the temporal coexistence of alcohol use and emotional changes does not establish that alcohol caused those psychological responses, particularly when both are assessed simultaneously [14,15].

Previous studies have documented associations between sporting events and individual psychological or behavioral outcomes, including anxiety, stress, emotional responses, and alcohol consumption. Therefore, the contribution of the present study does not lie in examining these individual associations in isolation, but rather in the simultaneous assessment of multiple psychological and health-related behavioral domains within the same population and within a tightly defined temporal window surrounding a specific major international sporting event. This approach allows potentially divergent patterns across psychological and behavioral domains to be examined concurrently.

Sexual attitudes were additionally included as an exploratory domain within this multidimensional assessment. Rather than assuming that a single sporting event would modify relatively stable erotophilia–erotophobia orientations, this measure was included to examine whether sexual-attitude scores differed between two closely spaced assessment periods surrounding an emotionally salient naturalistic event. Given the limited theoretical and empirical evidence supporting short-term variation in this construct in relation to sporting events, analyses involving EROS were considered exploratory and hypothesis-generating.

Much of the available evidence is based on retrospective reports, physiological monitoring during a match, population-level outcomes, or assessments conducted only after the sporting event [16,17]. Consequently, fewer studies have evaluated the same population both before and after a single [18], clearly defined international soccer match while simultaneously examining multiple psychological and health-related behavioral domains [19,20].

The 2026 FIFA World Cup provided a unique naturalistic context for examining psychological and behavioral patterns within a tightly defined temporal window surrounding a highly salient international sporting event. The Mexico–England match represented a particularly relevant occasion for Mexican spectators because it combined national identification, competitive uncertainty, and widespread social engagement.

Accordingly, the primary objective of this study was to characterize multidimensional psychological and health-related behavioral patterns observed around a major international soccer match by evaluating anxiety-related symptoms, depressive-related symptoms, subjective emotional well-being, and recent alcohol consumption across the pre-event and post-event assessment periods. Sexual attitudes were additionally examined as an exploratory domain. A secondary objective was to explore sex-stratified patterns and sociodemographic and behavioral factors associated with psychological outcomes and recent alcohol consumption, including an exploratory analysis of relatively higher EROS scores. We hypothesized that the principal psychological and health-related behavioral domains would exhibit distinct patterns between the pre-event and post-event assessment periods surrounding the sporting event. Specifically, we expected anxiety-related symptoms, emotional well-being, depressive-related symptoms, and recent alcohol consumption to show different patterns across the two assessment periods. No directional hypothesis was specified for the exploratory EROS analysis.

## 2. Materials and Methods

### 2.1. Study Design and Setting

This observational analytical study evaluated psychological and behavioral outcomes at two predefined assessment periods surrounding the Mexico–England match during the 2026 FIFA World Cup. Although the same cohort of participants completed both assessments, individual responses could not be linked across assessment periods because no personal identifiers or anonymous linkage codes were collected. Consequently, the study was analyzed as repeated cross-sectional marginal comparisons of the same underlying cohort rather than as a conventional paired longitudinal before-and-after study.

The study was conducted in Colima, Mexico, among students enrolled in a semi-schooled Sunday educational program. All participants attended the same educational institution and followed an identical academic schedule, attending face-to-face classes every Sunday from 09:00 to 15:00 h. To minimize environmental variability, both assessments were conducted in the same classroom under standardized conditions, including white artificial lighting, a room temperature of approximately 25 °C maintained by air conditioning, minimal external noise, and groups of approximately 30 students.

The pre-match assessment was performed approximately 10 h before the scheduled kick-off of the Mexico–England FIFA World Cup match during a specially scheduled academic session. The post-match assessment was conducted approximately 11 h after the end of the match during the first scheduled academic session on the following morning. Although the kick-off was delayed by approximately one hour because of adverse weather conditions, the interval between completion of the match and the post-match assessment remained within the first 12 h after the event, providing a temporally proximal post-event assessment.

The soccer match constituted a naturally occurring exposure shared by all participants. To maximize the likelihood that the event represented a meaningful emotional stimulus, participants were required to report interest in the FIFA World Cup match, support the Mexican national soccer team, and confirm that they had watched the entire match. Students who did not watch the complete match were excluded from the study. However, the degree of soccer fandom or emotional identification with the national team was not formally quantified and should be considered in future research, as psychological responses may differ according to spectator involvement [21].

### 2.2. Participants

Eligible participants were students aged 18 years or older enrolled in the Sunday semi-schooled educational program. Recruitment was performed through announcements during regular academic sessions one week before the FIFA World Cup match. Participation was voluntary, anonymous, and without financial compensation. Inclusion criteria (Participants were eligible if they: (1) were ≥18 years of age; (2) were officially enrolled in the Sunday educational program; (3) attended both institutional assessment sessions; (4) watched the complete Mexico–England FIFA World Cup match, and (5) provided written informed consent. Exclusion criteria (Participants were excluded if they: (1) reported a previous psychiatric disorder under active pharmacological treatment; (2) presented cognitive impairment preventing questionnaire completion; (3) submitted incomplete questionnaires for any primary study variable; and (4) reported not having watched the complete soccer match.

To preserve participant anonymity, no personal identifiers or anonymous linkage codes were collected. Consequently, although individual observations could not be paired, the pre-event and post-event assessments originated from the same underlying cohort and were analyzed as repeated cross-sectional marginal comparisons [22]. After applying eligibility criteria and data cleaning procedures, 146 observations were available for each assessment period, yielding a total analytical dataset of 292 observations.

### 2.3. Measures

A structured, self-administered questionnaire was used to collect sociodemographic characteristics, lifestyle behaviors, psychological outcomes, sexual attitudes, and recent health-related habits. The questionnaire comprised approximately 75 items, including investigator-developed questions and three validated psychometric instruments. Information collected included age, sex, employment status, physical activity, sports participation, smoking history, habitual alcohol consumption, breakfast habits, protein intake at breakfast, daily beverage consumption, social media use, self-perceived health, chronic diseases, socioeconomic status, and other behavioral variables. Psychological outcomes were assessed using the Hospital Anxiety and Depression Scale (HADS; 14 items), subjective emotional well-being using the World Health Organization Five Well-Being Index (WHO-5; 5 items), and sexual attitudes using the Revised Sexual Opinion Survey (R-SOS; 20 items) [10,23,24,25,26].

All questionnaires were administered under standardized classroom conditions during both assessment sessions. Participants completed the instruments in groups of approximately 30 students in a large, enclosed, air-conditioned classroom under white artificial lighting, with the ambient temperature maintained at approximately 25 °C. The environment remained quiet throughout data collection, and participants completed the questionnaires individually without interruptions or interaction with classmates.

Both assessments were conducted at approximately 09:00 h under comparable environmental conditions to minimize potential circadian and environmental influences. Participants completed the questionnaires before any academic activities were initiated, and the average completion time was approximately 30 min. Participants were instructed to maintain their usual morning routine before both assessments, including their habitual breakfast, and breakfast-related variables were recorded to characterize baseline nutritional conditions rather than to standardize dietary intake.

#### 2.3.1. Sociodemographic and Lifestyle Characteristics

Participants completed a structured questionnaire designed to collect demographic, socioeconomic, lifestyle, and health-related information. Sociodemographic variables included age, sex (male/female), marital status, employment status, head-of-household status, religious affiliation, and membership in an indigenous community. Socioeconomic status was classified according to the criteria established by the Mexican Association of Market Intelligence and Opinion Agencies (AMAI), which categorizes households into six socioeconomic levels based on housing characteristics, household assets, educational attainment, and living conditions: A/B (high), C+ (upper-middle), C (middle), D+ (lower-middle), D (low), and E (very low) socioeconomic status [27,28].

Lifestyle variables included regular sports participation, general physical activity, smoking history, habitual alcohol consumption, alcohol consumption frequency, and self-reported history of serious illness. These variables were collected because previous studies have suggested that socioeconomic conditions, lifestyle behaviors, and health status may influence psychological well-being and emotional responses in young adults.

In addition, participants reported recent nutritional and beverage consumption before each assessment. Specifically, they indicated whether they had consumed breakfast, protein-containing foods, and the type of beverage consumed during the previous eight hours. Beverage categories included water, coffee, juice, tea, flavored beverages, energy drinks, and alcoholic beverages. Recent alcohol consumption during the eight hours preceding each assessment was additionally recorded as a dichotomous variable (Yes/No) and was considered one of the main behavioral outcomes of interest.

Habitual alcohol consumption and recent alcohol consumption were analyzed separately. Habitual alcohol use was considered a baseline characteristic, whereas recent alcohol consumption during the eight hours preceding each assessment was evaluated as a temporally proximal behavioral measure. Because the post-event assessment was conducted approximately 11 h after the match, the eight-hour recall window did not encompass the entire interval following the sporting event and should not be interpreted as representing alcohol consumption during the eight hours immediately after the match.

#### 2.3.2. Mental Health: Anxiety and Depression

Symptoms of anxiety and depression were assessed using the validated Spanish version of the Hospital Anxiety and Depression Scale (HADS), one of the most frequently used screening instruments for psychological distress in community and non-clinical populations. The questionnaire comprises 14 items equally distributed into two seven-item subscales evaluating anxiety (HADS-A) and depression (HADS-D), with scores ranging from 0 to 21 for each domain.

At both assessment sessions, participants were explicitly instructed to answer the HADS items according to how they felt at the time of questionnaire completion. The standardized temporal instruction was: “Please answer each item according to how you feel right now, at this moment.” This instruction was applied consistently during the pre-event and post-event assessments to contextualize responses to the participants’ current state. However, this modified temporal administration of the HADS has not been specifically validated for detecting variation over an interval of approximately 24 h. Therefore, differences between assessment periods were interpreted cautiously as differences in HADS scores under the study-specific administration conditions, rather than as validated acute changes in anxiety or depressive symptomatology.

Continuous scores were analyzed as primary outcomes to evaluate differences between the pre-event and post-event assessments. In addition, clinically relevant symptomatology was explored using previously validated screening thresholds of HADS-A ≥ 8 for anxiety-related symptoms and HADS-D ≥ 7 for depressive-related symptoms. These thresholds identify participants with clinically relevant symptom burden and should not be interpreted as psychiatric diagnoses. Consequently, throughout this manuscript, the terms anxiety-related symptoms and depressive-related symptoms are used rather than diagnostic terminology [10].

#### 2.3.3. Subjective Emotional Well-Being

Subjective emotional well-being was assessed using the World Health Organization-Five Well-Being Index (WHO-5), a validated self-report instrument widely used to evaluate positive emotional well-being in both general and young adult populations. The WHO-5 consists of five positively worded items scored on a six-point Likert scale. Raw scores range from 0 to 25 and were multiplied by four to obtain a standardized score ranging from 0 to 100, with higher scores indicating better emotional well-being [29,30].

Continuous WHO-5 scores were analyzed as primary outcomes to compare emotional well-being between the pre-event and post-event assessments. For multivariable analyses, participants were additionally classified according to the recommended screening threshold of 50 points, with scores ≤ 50 indicating reduced emotional well-being and scores > 50 indicating preserved emotional well-being. This cut-off has been extensively validated for screening reduced well-being and identifying individuals who may require further evaluation for depressive-related symptoms, although it is not intended to establish a clinical diagnosis [31,32]. The original WHO-5 assesses well-being using a two-week reference period. In the present study, the five original items, response options, and scoring procedure were retained; however, the administration instructions were slightly contextualized to ask participants to respond according to how they were feeling at the time of each assessment. This modification was intended to align the instrument with the temporally proximal assessment design. Because this adapted temporal instruction has not been specifically validated for detecting changes over an approximately 24 h interval, WHO-5 differences were interpreted as assessment-period differences rather than as validated acute changes in emotional well-being [33,34].

#### 2.3.4. Sexual Attitudes (Erotophilia–Erotophobia)

Sexual attitudes were assessed using the 20-item Spanish-language Encuesta Revisada de Opinión Sexual (EROS), developed by Del Río Olvera et al. through adaptation and psychometric refinement of the Sexual Opinion Survey. This 20-item instrument assesses the erotophilia–erotophobia continuum and demonstrated adequate item–total correlations and internal consistency in its original Spanish psychometric evaluation. Items are rated on a seven-point Likert scale, with higher total scores indicating more erotophilic attitudes toward sexual stimuli [26].

In this study, EROS scores were analyzed primarily as continuous variables. For the exploratory post-event regression analysis, scores were additionally dichotomized at the sample median (26 points), with scores > 26 representing a relatively greater erotophilic tendency and scores ≤ 26 representing a relatively greater erotophobic tendency [35,36]. This median-based categorization was used solely for exploratory analytical purposes and does not represent a validated clinical or diagnostic cutoff. Higher scores reflect a more positive, open, and comfortable orientation toward sexuality, whereas lower scores reflect relatively more avoidant or negative attitudes [36].

#### 2.3.5. Lifestyle Behaviors and Substance Use

Lifestyle characteristics included regular sports participation, habitual physical activity, smoking history, and alcohol consumption. Particular emphasis was placed on recent alcohol consumption during the eight hours preceding each assessment, given the social context surrounding the FIFA World Cup match [37,38,39].

Participants also reported the principal beverage consumed during the previous eight hours. These data were used to characterize differences in beverage consumption patterns between assessment periods, with particular emphasis on alcohol consumption. For inferential analyses, recent alcohol intake was analyzed as a dichotomous variable (Yes/No), whereas beverage categories were summarized descriptively. The quantity of alcohol consumed, drinking duration, binge-drinking patterns, and level of intoxication were not assessed.

Regular sports participation, physical activity, smoking history, and recent alcohol consumption were subsequently included as explanatory variables in multivariable logistic regression models to evaluate their independent associations with clinically relevant anxiety-related symptoms, depressive-related symptoms, emotional well-being, and sexual attitudes.

### 2.4. Ethical Considerations

The study was conducted in accordance with the ethical principles of the Declaration of Helsinki and complied with all applicable national regulations governing research involving human participants. Ethical approval was obtained from the Research Ethics Committee of the State Cancer Institute and the University of Colima (Approval No. CEICANCL230317-ASEXCCC-06).

The present study was conducted as an authorized scientific extension of the original protocol. The corresponding scientific extension was formally authorized and documented by the Research Ethics Committee on 3 July 2026. The Committee is registered under No. 06-CEI-001-20200721. This extension specifically covered the analysis of psychological variables, emotional well-being, sexual attitudes, recent alcohol consumption, and sociodemographic and behavioral characteristics assessed in students within the temporal context of an international sporting event. The Research Ethics Committee determined that this extension did not substantially modify the study population, eligibility criteria, recruitment procedures, confidentiality safeguards, or level of participant risk and therefore did not require a new independent ethical review.

All participants provided written informed consent before enrollment. Participation was voluntary, anonymous, and without financial compensation. To ensure complete confidentiality, no personally identifiable information or anonymous linkage codes were collected. Although the same participants completed both assessments, individual responses could not be matched across assessment periods. Consequently, within-participant changes and correlations could not be estimated, and paired statistical analyses could not be performed.

Anonymous linkage codes were not incorporated into the original data-collection procedure because the protocol prioritized complete anonymity and did not anticipate the subsequent analytical importance of estimating within-participant changes. In retrospect, the use of non-identifiable linkage codes would have preserved participant confidentiality while permitting paired analyses. Their absence therefore represents an important methodological limitation of the present study and was explicitly considered when interpreting comparisons between assessment periods. Throughout the study and its subsequent extension, confidentiality, anonymity, protection of personal data, and respect for participants’ rights were maintained in accordance with the ethical principles established in the original approval.

### 2.5. Statistical Analysis

Statistical analyses were performed using IBM SPSS Statistics version 28.0 (IBM Corp., Armonk, NY, USA) [40].

Continuous variables were summarized as means ± standard deviations (SD), medians, ranges, and interquartile ranges (IQR), whereas categorical variables were expressed as frequencies and percentages [41]. The distribution of continuous variables was evaluated using the Kolmogorov–Smirnov test, which indicated that most psychological scale scores deviated from a normal distribution.

Because individual responses could not be linked across the two assessment periods, paired statistical procedures could not be performed. Comparisons were therefore conducted at the marginal assessment-period level using the Mann–Whitney U test for continuous variables and Pearson’s chi-square test or Fisher’s exact test for categorical variables, as appropriate. Odds ratios (ORs) with 95% confidence intervals (CIs) were calculated for relevant categorical comparisons. Importantly, because both assessments originated from the same underlying cohort, the independence assumption underlying these tests cannot be considered fully satisfied. Accordingly, these analyses were treated as repeated cross-sectional marginal comparisons and their inferential results were interpreted cautiously. They should not be considered equivalent to paired longitudinal analyses and do not permit estimation of within-participant change.

Additional analyses were stratified by sex to explore potential sex-specific patterns. These analyses were considered exploratory because of the smaller subgroup sample sizes and reduced statistical precision. Formal sex × predictor interaction terms were not fitted; therefore, differences in statistical significance between sex-stratified models were not interpreted as evidence of effect modification by sex.

Multivariable binary logistic regression analyses were performed using post-event observations only (*n* = 146) to identify factors associated with clinically relevant anxiety-related symptoms (HADS-A ≥ 8), depressive-related symptoms (HADS-D ≥ 7), high emotional well-being (WHO-5 > 50), and relatively higher EROS scores [10,36,42]. Pre-event and post-event observations were not combined in these models because individual responses could not be linked across assessment periods. Consequently, assessment period was not included as a predictor, and sex × assessment-period interaction terms were not applicable to these post-event-only regression models.

EROS was analyzed primarily as a continuous variable to preserve the full information contained in the scale. For an exploratory post-event logistic regression analysis, EROS scores were additionally dichotomized at the sample median (26 points), with scores > 26 representing a relatively greater erotophilic tendency and scores ≤ 26 representing a relatively greater erotophobic tendency. This categorization was used solely to facilitate exploratory estimation of odds ratios and does not represent a validated diagnostic or clinical cutoff. Because the two categories are complementary, only the relatively higher EROS category was modeled as the binary outcome.

For the psychological-outcome models, sex, employment status, smoking history, regular sports participation, and recent alcohol consumption were included as prespecified covariates based on their conceptual and epidemiological relevance. All covariates were entered simultaneously into each model; no automated stepwise or significance-based variable-selection procedure was used. Adjusted odds ratios (aORs) with 95% CIs were reported.

A separate multivariable logistic regression model was constructed with recent alcohol consumption as the dependent variable. Employment status, regular sports participation, anxiety-related symptoms, and depressive-related symptoms were entered simultaneously as prespecified explanatory variables. Because alcohol consumption and psychological symptoms were assessed during the same post-event assessment, the resulting associations were interpreted as cross-sectional, and no temporal or causal direction was inferred.

Multicollinearity among predictors was assessed using variance inflation factors (VIFs), with values < 5 considered indicative of no problematic multicollinearity. Model calibration was evaluated using the Hosmer–Lemeshow goodness-of-fit test, with *p* > 0.05 interpreted as no evidence of lack of fit.

The overall comparisons of anxiety-related symptoms, depressive-related symptoms, emotional well-being, and recent alcohol consumption between assessment periods were considered the principal analyses. Analyses of sexual attitudes, sex-stratified comparisons, multivariable regression models, and sex-stratified regression analyses were considered secondary or exploratory [42]. No formal adjustment for multiple testing was applied; therefore, *p*-values from secondary and exploratory analyses were considered nominal and interpreted cautiously because of the increased risk of type I error. For these analyses, interpretation emphasized effect sizes and 95% confidence intervals rather than statistical significance alone [42,43].

All statistical tests were two-sided. For the principal analyses, a *p*-value < 0.05 was considered statistically significant. *p*-values from secondary and exploratory analyses were considered nominal and interpreted cautiously in the context of multiple testing.

#### Sample Size

No formal a priori sample-size calculation was performed. The sample size was determined by the number of eligible participants from the study population who agreed to participate and completed both assessment sessions, in accordance with recommendations to transparently report how the final study size was determined [44]. A total of 146 participants completed both the pre-event and post-event assessments and constituted the final study sample.

Given the fixed sample size, multivariable and particularly sex-stratified analyses were considered exploratory and interpreted cautiously, with emphasis on the magnitude and precision of the estimated associations and their 95% confidence intervals. For logistic regression analyses, model precision depends not only on the total sample size but also on the number of outcome events relative to the number of predictor parameters included in each model [45,46]. Accordingly, the analytical sample size and number of outcome events were reported for the multivariable models, and the sex-stratified models were interpreted with particular caution because of their smaller subgroup sample sizes and reduced number of outcome events.

## 3. Results

**Figure 1:**
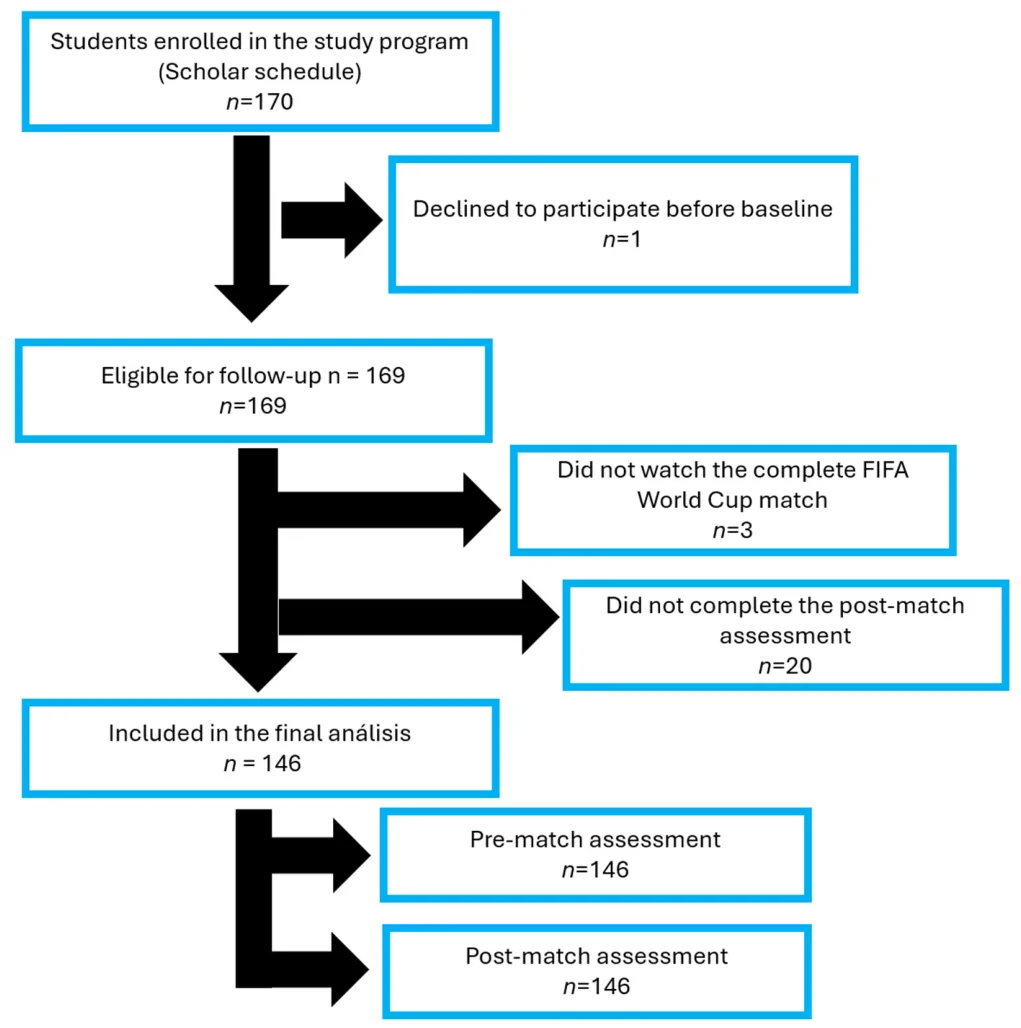
Participant Selection Flowchart: Invitation, Inclusion, and Exclusion. Of the 170 students enrolled in the Sunday educational program, 24 were excluded because they did not attend one or both assessment sessions (*n* = 20), did not watch the complete FIFA World Cup match (*n* = 3), or declined to participate (*n* = 1). A total of 146 students met all eligibility criteria and completed both assessments under standardized classroom conditions.

The participant flow is summarized in Figure 1. A total of 170 students enrolled in the Sunday educational program were invited to participate. One student declined participation before the baseline assessment, and three students were excluded because they did not watch the complete Mexico–England FIFA World Cup match, which was an inclusion criterion for the post-match assessment. Therefore, 166 students were eligible for inclusion. Of these, 20 did not complete one of the two scheduled assessment sessions and were excluded from the final analysis because complete data from both time points were required. Consequently, the final analytical sample consisted of 146 students who completed both assessments under standardized classroom conditions.

In socioeconomic terms, the sample was predominantly composed of students from low socioeconomic backgrounds, with the largest proportions classified in the very low (AMAI level E, 25.9%) and low (AMAI level D, 23.1%) categories. Additional participants belonged to the lower-middle (D+, 16.3%) and upper-middle (C+, 19.0%) socioeconomic levels, whereas no participants were classified in the highest AMAI category (A/B). Regarding religious affiliation, Catholicism was the most prevalent (45.6%), followed by Christianity (25.2%), whereas nearly one-quarter of the students reported no religious affiliation (24.5%). Indigenous community membership was uncommon (5.4%) (Table 1).

Regular sports participation and the types of sports practiced were also descriptively examined according to sex. These data are provided as Supplementary Descriptive Information on the characteristics of the study population (Table S1).

### 3.1. Differences in Psychological Outcomes Between Pre-Event and Post-Event Assessments

Table 2 summarizes the comparison of psychological outcomes assessed before and after the Mexico–England FIFA World Cup match. Anxiety-related symptom scores were significantly lower at the post-event assessment than at the pre-event assessment (mean ± SD: 9.00 ± 3.68 vs. 6.12 ± 4.18; Mann–Whitney U = 6520.0, Z = −5.752, *p* < 0.001), corresponding to a moderate effect size (*r* = 0.34). No statistically significant difference in depressive-related symptom scores was observed between the pre-event and post-event assessments (6.41 ± 3.93 vs. 6.77 ± 3.68; *p* = 0.420).

Subjective emotional well-being was lower at the post-event assessment (48.90 ± 11.80 vs. 44.10 ± 11.50; Mann–Whitney U = 8003.5, Z = −3.703, *p* < 0.001), with a small-to-moderate effect size (*r* = 0.22).

EROS scores were slightly lower at the post-event assessment than at the pre-event assessment (25.25 ± 4.10 vs. 26.19 ± 3.50; Mann–Whitney U = 9123.5, Z = −2.135, *p* = 0.033), with a small effect size (*r* = 0.12). Given the exploratory nature of this outcome and the absence of formal multiplicity adjustment, this finding should be interpreted cautiously. Overall, differences between the pre-event and post-event assessments were observed for anxiety-related symptoms and emotional well-being, whereas depressive-related symptoms remained relatively stable. The small difference observed in sexual attitudes was considered exploratory and of limited practical significance.

### 3.2. Differences in Beverage Consumption Between Assessment Periods

Beverage consumption patterns changed substantially between the pre-event and post-event assessments (Table 3). Before the soccer match, water was the predominant beverage, being reported by 67.8% of participants, followed by coffee (21.2%), whereas alcohol consumption during the previous eight hours was uncommon (0.7%). At the post-event assessment, alcohol was the most frequently reported beverage (41.1%), while water consumption decreased markedly to 19.2%. Smaller increases were also observed for juice (4.1% vs. 12.3%) and energy drink consumption (3.4% vs. 5.5%).

Consistent with these findings, recent alcohol consumption was substantially more frequent at the post-event assessment. Only one participant (0.7%) reported alcohol intake during the eight hours preceding the pre-match assessment compared with 60 participants (41.1%) at the post-event assessment (χ^2^ = 72.14, *p* < 0.001).

**Figure 2:**
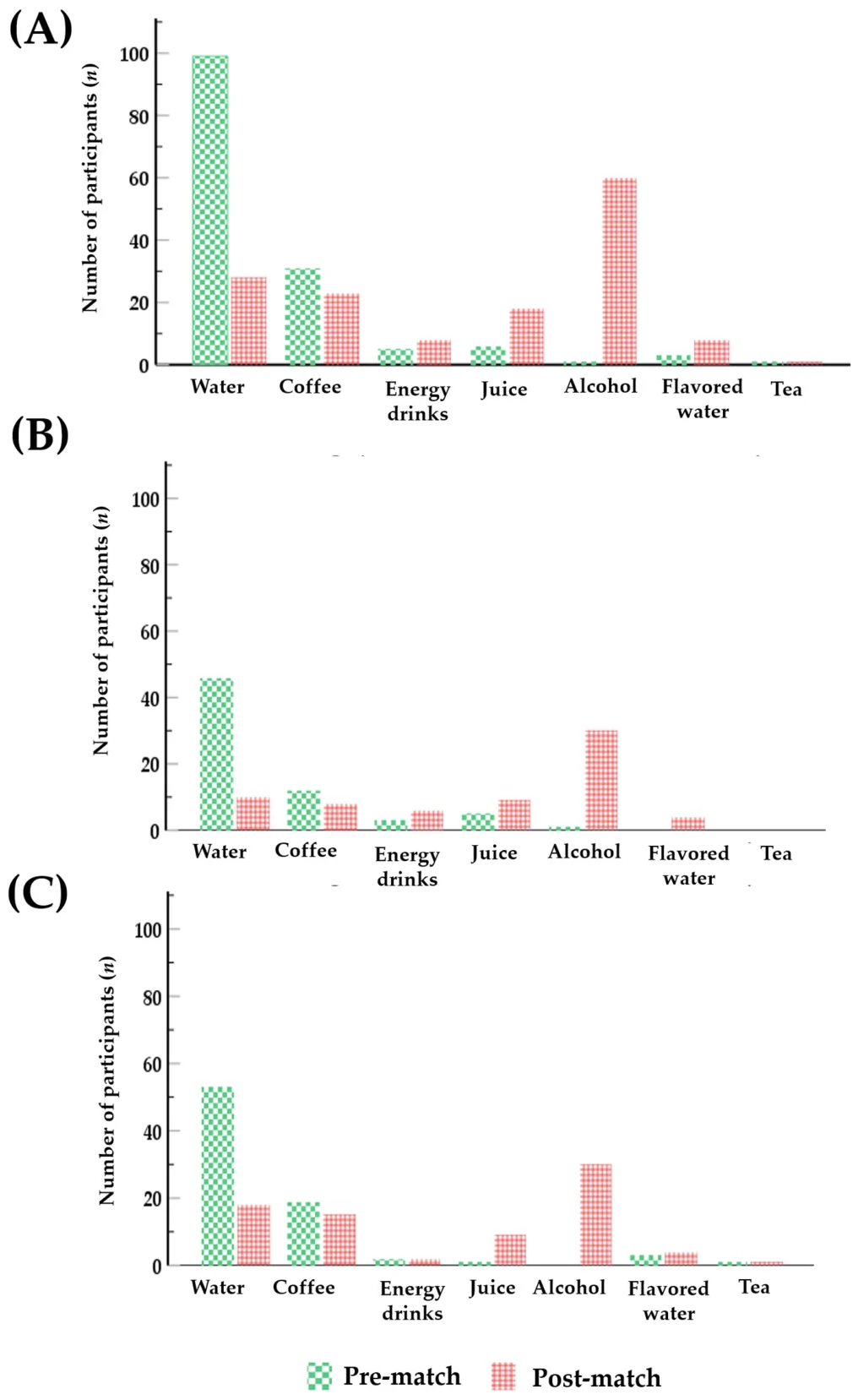
Beverage consumption during the 8 h preceding the pre-event and post-event assessments. (**A**) Overall beverage consumption. (**B**) Beverage consumption among men. (**C**) Beverage consumption among women. Water was the predominant beverage reported at the pre-event assessment in both sexes, whereas alcohol was the most frequently reported beverage at the post-event assessment, accompanied by a lower frequency of reported water consumption.

As illustrated in Figure 2, similar beverage consumption patterns were observed among both men and women. Water was the predominant beverage before the match in both sexes, whereas alcohol became the most frequently reported beverage after the match, accounting for 44.8% of beverages consumed by men and 38.0% by women. These findings indicate that the higher frequency of recent alcohol consumption at the post-event assessment was observed in both sexes.

### 3.3. Sex-Stratified Differences in Psychological Outcomes

Sex-stratified analyses showed lower anxiety-related symptom scores at the post-event assessment in both men and women (Table 4). Among men, mean scores were 7.84 ± 3.21 at the pre-event assessment and 4.67 ± 3.85 at the post-event assessment (*p* < 0.001, *r* = 0.42). Among women, the corresponding scores were 9.99 ± 3.78 and 7.35 ± 4.07 (*p* < 0.001, *r* = 0.30).

Emotional well-being scores were also lower at the post-event assessment in both men (47.12 ± 12.32 vs. 43.28 ± 11.21; *p* = 0.036) and women (50.36 ± 11.12 vs. 44.88 ± 11.80; *p* = 0.002, *r* = 0.25). No statistically significant differences in depressive-related symptom scores were observed between assessment periods in either sex.

### 3.4. Factors Associated with Psychological Outcomes

In the post-event multivariable analyses (Table 5), female sex was associated with higher odds of clinically relevant anxiety-related symptoms (aOR = 2.78, 95% CI: 1.28–6.04; *p* = 0.010). Regular sports participation was associated with lower odds of clinically relevant depressive-related symptoms (aOR = 0.23, 95% CI: 0.11–0.49; *p* < 0.001). No statistically significant associations were observed for high emotional well-being or relatively higher EROS scores. The EROS model was considered exploratory, and none of the evaluated covariates was significantly associated with relatively higher EROS scores. Given the secondary or exploratory nature of the multivariable analyses and the absence of formal multiplicity adjustment, these estimates should be interpreted cautiously, with emphasis on effect sizes and confidence intervals rather than statistical significance alone.

### 3.5. Factors Associated with Alcohol Consumption Following the Soccer Match

In the separate post-event model of recent alcohol consumption (*n* = 146; 60 outcome events), employment was associated with lower odds of reporting alcohol consumption during the preceding eight hours (aOR = 0.29, 95% CI: 0.14–0.58; *p* < 0.001). Regular sports participation showed an imprecise positive association that did not reach the conventional significance threshold (aOR = 1.95, 95% CI: 0.91–4.19; *p* = 0.085), whereas anxiety-related and depressive-related symptom categories were not significantly associated with recent alcohol consumption (Table 6). These associations are cross-sectional and should not be interpreted as indicating temporal or causal relationships.

Exploratory sex-stratified multivariable analyses were performed to examine potential subgroup-specific patterns associated with recent alcohol consumption (Table 7). The analyses included 67 men and 79 women, with 30 alcohol-consumption events in each subgroup. Given the limited number of outcome events relative to the number of predictors, these analyses were considered exploratory and the resulting estimates should be interpreted cautiously.

Among men, employment was associated with lower odds of recent alcohol consumption (aOR = 0.22, 95% CI: 0.08–0.64; *p* = 0.005). Regular sports participation, anxiety-related symptoms, and depressive-related symptoms were not significantly associated with recent alcohol consumption in the male subgroup.

Among women, employment was also associated with lower odds of recent alcohol consumption (aOR = 0.28, 95% CI: 0.10–0.80; *p* = 0.017). Regular sports participation was associated with higher odds of recent alcohol consumption (aOR = 4.13, 95% CI: 1.24–13.71; *p* = 0.021), although the wide confidence interval indicates substantial statistical imprecision. Anxiety-related and depressive-related symptoms were not significantly associated with recent alcohol consumption among women.

These subgroup-specific findings are exploratory and were not adjusted for multiple testing. Moreover, statistical significance within one sex and lack of statistical significance within the other should not be interpreted as evidence of effect modification by sex in the absence of a formal interaction test.

## 4. Discussion

The present study identified multidimensional differences in psychological and behavioral outcomes between the pre-event and post-event assessment periods surrounding a major international soccer match. Anxiety-related symptom scores were lower at the post-event assessment, subjective emotional well-being was also lower, depressive-related symptoms remained relatively stable, and recent alcohol consumption was substantially more frequent. These findings indicate that different psychological and behavioral domains exhibited distinct patterns between assessment periods. However, because several co-occurring exposures were not measured, these differences cannot be attributed specifically to the soccer match.

Rather than supporting the common assumption that major sporting events produce a single, homogeneous emotional response, our findings indicate that anxiety-related symptoms, emotional well-being, depressive-related symptoms, and health-related behaviors exhibited distinct patterns across the two assessment periods surrounding the same soccer match. To our knowledge, few studies have simultaneously evaluated anxiety-related symptoms, depressive-related symptoms, subjective emotional well-being, sexual attitudes, and alcohol consumption immediately before and after the same FIFA World Cup match using standardized instruments under controlled classroom conditions. This multidimensional approach provides a more comprehensive characterization of psychological and behavioral patterns observed within a tightly defined temporal window surrounding a major sporting event than studies focusing on isolated outcomes.

One of the principal findings was that anxiety-related symptom scores were lower at the post-event assessment than at the pre-event assessment. Previous studies have shown that anticipation of emotionally salient or uncertain events may be accompanied by heightened psychological and physiological arousal [47]. Resolution of uncertainty has therefore been proposed as one potential explanation for lower anxiety following such events [18,47,48]. However, uncertainty was not directly measured in the present study, and the observed assessment-period difference cannot establish that resolution of uncertainty, or the soccer match itself, accounted for the lower post-event HADS-A scores.

An apparently paradoxical finding of the present study was the observation that both anxiety-related symptom scores and subjective emotional well-being scores were lower at the post-event assessment. Although these findings may initially appear contradictory, they likely reflect different psychological constructs. Anxiety measured by the HADS primarily captures anticipatory tension and autonomic activation, whereas the WHO-5 evaluates positive emotional functioning, vitality, and subjective psychological well-being rather than psychological distress [10,34]. Several unmeasured contextual factors could potentially contribute to these assessment-period patterns, including uncertainty surrounding the event, sleep disruption, fatigue, match satisfaction or disappointment, recent alcohol consumption, and changes in daily routines. Because these factors were not directly measured, their respective contributions cannot be determined from the present data [7,49].

However, the lower WHO-5 scores observed at the post-event assessment should not be interpreted as a direct psychological effect of the soccer match. Because the post-event evaluation was conducted the following morning, the observed difference in emotional well-being may also reflect several co-occurring factors, including sleep loss or disruption, fatigue, recent alcohol consumption, altered routine, emotional investment in the match, match satisfaction or disappointment, and hydration status. Sleep loss and disruption are particularly relevant because experimental and meta-analytic evidence indicates that insufficient sleep can reduce positive affect and impair emotional functioning [50]. Moreover, previous research in soccer contexts suggests that match outcomes can be associated with short-term variations in subjective well-being and emotional responses [51,52]. These factors were not measured and therefore could not be disentangled from the temporal context of the sporting event [50,51,52]. Accordingly, the WHO-5 findings should be interpreted as differences between assessment periods rather than as evidence of a match-specific effect on emotional well-being.

Depressive-related symptoms remained relatively stable between the pre-event and post-event assessments. This observation is consistent with the expectation that depressive symptomatology may be less responsive than anxiety-related symptoms to very short-term situational variation.

A small difference in continuous EROS scores was observed between assessment periods, with slightly lower scores at the post-event assessment. However, the effect size was small (*r* = 0.12), and this outcome was examined within a broader set of secondary and exploratory analyses without formal multiplicity adjustment. Therefore, the possibility of a chance finding cannot be excluded, and this result should be considered hypothesis-generating rather than evidence of a meaningful event-related change in sexual attitudes. Consistent with this cautious interpretation, the exploratory post-event regression analysis did not identify significant associations between relatively higher EROS scores and the evaluated sociodemographic or behavioral covariates. Further studies specifically designed to examine sexual-attitude variation around emotionally salient events are needed to determine whether this observation is reproducible [53,54,55].

One of the most pronounced behavioral differences was the substantially higher frequency of recent alcohol consumption at the post-event assessment. Whereas almost no participants reported alcohol intake during the eight hours preceding the pre-event assessment, more than two-fifths reported recent alcohol consumption at the post-event assessment. Although this pattern occurred in the temporal context of the soccer match, the study design does not permit attribution of the difference specifically to the sporting event. Previous studies have similarly reported higher alcohol consumption in the context of internationally relevant sporting events, suggesting that these occasions may coincide with short-term variations in health-related behaviors among young adults [56]. An additional consideration is that all participants supported the Mexican national team, which was defeated by England. Therefore, the findings correspond specifically to assessments surrounding an unfavorable sporting outcome, and whether similar patterns would be observed after a victory or draw remains unknown. However, because alcohol consumption was assessed only as a binary variable, the data cannot distinguish modest social drinking from heavier or potentially hazardous consumption. Moreover, alcohol consumption and psychological symptoms were assessed concurrently; therefore, their associations do not establish temporal directionality.

Beyond the immediate psychological findings, the substantially higher frequency of recent alcohol consumption observed at the post-event assessment has important public health and safety implications. Major sporting events are consistently associated with increased alcohol availability and consumption, which may contribute to impaired judgment, interpersonal violence, traffic injuries, accidental trauma, sleep disruption, and other acute adverse health outcomes among spectators and surrounding communities [16,17]. Although the present study did not assess alcohol quantity or alcohol-related harms, the higher frequency of recent alcohol consumption observed at the post-event assessment highlights the relevance of further investigating alcohol-related behaviors surrounding major sporting events [57,58].

From a broader public-health perspective, sport-related well-being and resilience may be shaped not only by individual factors but also by participation environments, organizational responses, equity, and policy-level strategies, as highlighted by Ciaccioni et al. [59]. This multilevel perspective may inform preventive approaches surrounding major sporting events.

The post-event multivariable analyses identified several associations that warrant cautious interpretation. Female sex was associated with higher odds of clinically relevant anxiety-related symptoms, whereas regular sports participation was associated with lower odds of clinically relevant depressive-related symptoms. In the separate model of recent alcohol consumption, employment was associated with lower odds of reporting alcohol consumption during the preceding eight hours. These associations are consistent with previous literature linking sociodemographic and behavioral characteristics with mental health and alcohol-related behaviors; however, because the models were cross-sectional and considered secondary analyses, they should not be interpreted as demonstrating temporal or causal relationships [60,61,62].

**Figure 3:**
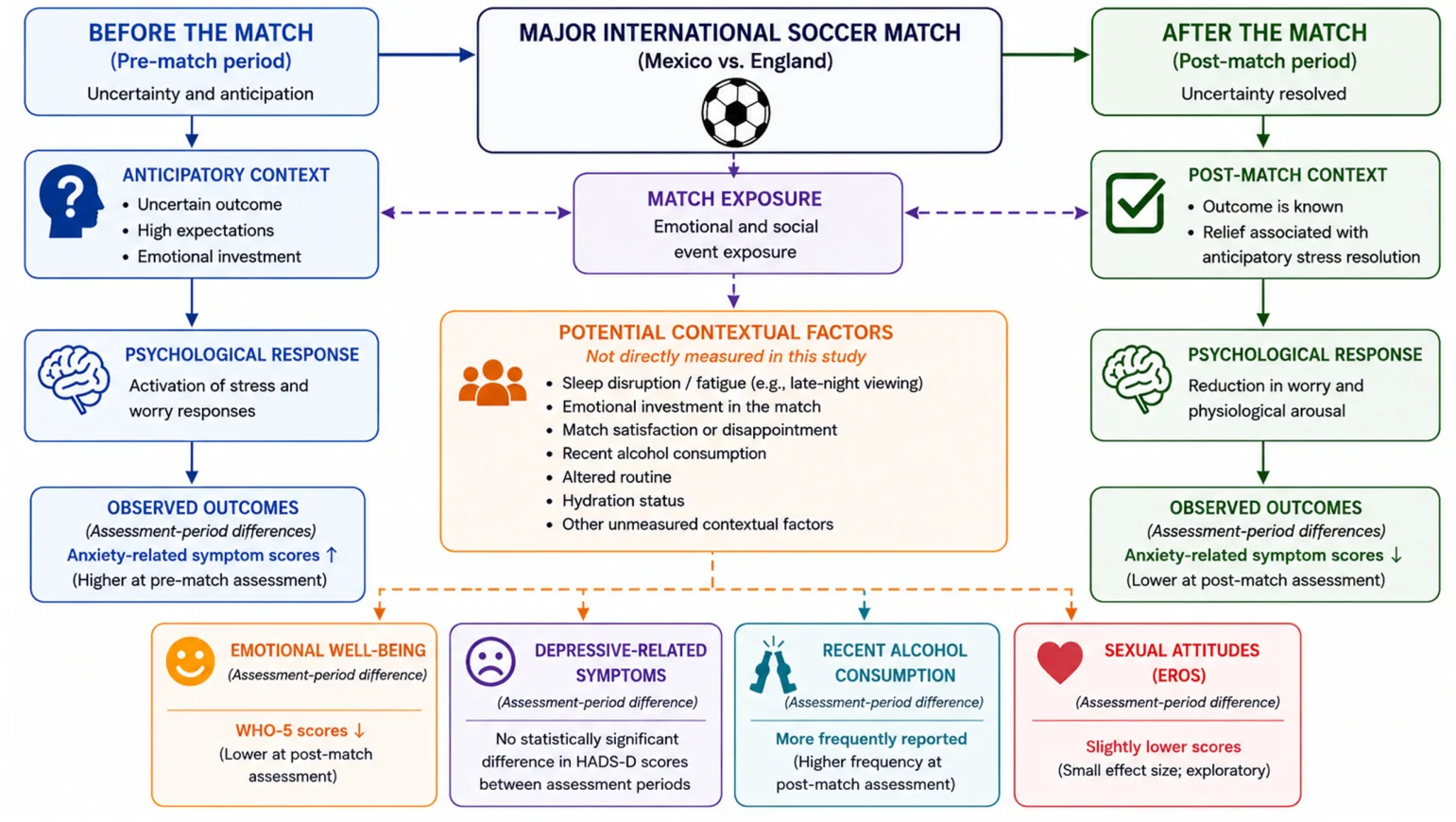
Hypothesis-generating conceptual framework illustrating the observed assessment-period differences and potential contextual factors surrounding the sporting event. Observed findings represent marginal differences between the pre-event and post-event assessment periods and should not be interpreted as within-participant changes or causal effects attributable to the event. Upward and downward arrows indicate higher and lower values or reporting frequencies, respectively, at the specified assessment period; they do not represent within-participant changes. Blue and green denote the pre-match and post-match contexts, respectively, whereas orange, purple, light blue, and red distinguish the outcome domains and do not indicate effect magnitude or statistical significance. Solid arrows indicate only the temporal sequence of the assessment periods and do not imply causality. Dashed arrows represent hypothesized relationships involving contextual factors that were not directly measured or statistically tested and are included solely to identify potential directions for future research.

Exploratory sex-stratified analyses showed that employment was associated with lower odds of recent alcohol consumption in both men and women. Among women, regular sports participation was associated with higher odds of recent alcohol consumption; however, the estimate was imprecise, as reflected by the wide confidence interval. No statistically significant associations between anxiety-related or depressive-related symptoms and recent alcohol consumption were observed within either sex-specific model. Given the limited number of outcome events, absence of multiplicity adjustment, and lack of formal interaction testing, these subgroup findings should be considered exploratory and should not be interpreted as evidence of sex-specific effects or effect modification [62,63]. These results therefore represent cross-sectional associations rather than causal relationships (Figure 3).

This study has several strengths. Assessments were performed within a tightly defined temporal window before and after the same internationally relevant soccer match under standardized classroom conditions using validated psychological instruments. The homogeneous educational environment and consistent assessment schedule minimized environmental variability and increased internal consistency.

Furthermore, the simultaneous evaluation of multiple psychological domains together with behavioral outcomes within the same population and a tightly defined temporal window provides a broader perspective on psychological and behavioral patterns observed around a major sporting event than assessments focused on a single domain [63,64]. An additional consideration is that the psychological responses observed in this study occurred after Mexico was defeated by England. Consequently, the findings should be interpreted within the context of an unfavorable sporting outcome for the supported national team. Emotional responses following major sporting events may differ when the supported team wins, loses, or draws, as well as according to the degree of team identification or spectator fandom. Because the present study did not quantify these factors, future research should examine whether the multidimensional psychological response identified here varies according to match outcome and individual emotional investment in the sport.

Several limitations should be acknowledged. First, although the same student population participated in both assessments, anonymous data collection prevented individual-level linkage. Consequently, paired analyses could not be performed, and the findings should be interpreted as repeated cross-sectional marginal differences between assessment periods rather than within-participant changes [62,63]. Second, although the original items, response options, and scoring procedures of the WHO-5 and HADS were retained, their administration instructions were contextualized to the participants’ state at the time of each assessment. This modification represents a departure from the standard two-week reference period of the WHO-5, while the current-state administration of the HADS has not been specifically validated for detecting short-term variation. These adapted temporal instructions have not been validated for detecting variation over an interval of approximately 24 h and may affect the temporal validity and comparability of the resulting scores. Accordingly, differences between assessment periods should not be interpreted as validated acute changes in the underlying psychological constructs. Third, participants were recruited from a single educational institution in western Mexico, and the study focused on a single FIFA World Cup match, which may limit the generalizability of the findings to other populations, sporting events, and contexts. Fourth, no formal a priori sample-size calculation was performed, and the sex-stratified regression analyses had limited statistical precision because of the relatively small subgroup sizes and number of outcome events. These subgroup analyses should therefore be considered exploratory and should not be interpreted as definitive evidence of sex-related differences. Fifth, multiple secondary and exploratory analyses were performed without formal adjustment for multiplicity, increasing the potential for type I error, particularly for findings with small effect sizes or marginal *p*-values. In addition, although EROS was retained as a continuous measure for the assessment-period comparison, median-based dichotomization was used for the exploratory logistic regression analysis. This approach facilitated estimation of odds ratios but entails loss of information and statistical power, and the resulting categories should not be interpreted as validated clinical or diagnostic classifications. Finally, the uncontrolled observational design precludes causal inference, and the observed assessment-period differences should not be interpreted as effects attributable specifically to the sporting event.

## 5. Conclusions

This study identified distinct psychological and health-related behavioral patterns between assessment periods surrounding a major international soccer match. Anxiety-related symptom scores and emotional well-being scores were lower at the post-event assessment, depressive-related symptom scores showed no significant difference, and recent alcohol consumption was more frequently reported. EROS scores were also slightly lower, although this finding had a small effect size and was exploratory. The simultaneous assessment of multiple domains within the same cohort and a tightly defined temporal window provides a multidimensional characterization of responses surrounding an emotionally salient sporting event. However, because individual observations could not be linked and the study lacked an unexposed comparison group, these findings represent marginal assessment-period differences and should not be interpreted as within-participant changes or causal effects of the match. Further longitudinal studies with linked observations and measurement of relevant contextual factors are warranted.

## Figures and Tables

**Table 1:** Sociodemographic Characteristics of the Study Sample.

Characteristic	Category	*n* (%) or Mean ± SD
Age (years)	Mean ± SD	20.38 ± 2.14
Sex	Male	67 (45.9)
	Female	79 (54.1)
Employment status	No	63 (43.2)
	Yes	83 (56.8)
Head of household	No	130 (89.0)
	Yes	16 (11.0)
Marital status	Single	104 (71.2)
	Married	11 (7.5)
	Cohabiting (common-law union)	14 (9.6)
	Other	17 (11.6)
Socioeconomic level (AMAI)	C+ (upper-middle)	27 (18.5)
	C (middle)	23 (15.8)
	D+ (lower-middle)	24 (16.4)
	D (low)	34 (23.3)
	E (very low)	38 (26.0)
Religious affiliation	None	36 (24.7)
	Catholic	66 (45.2)
	Christian	37 (25.3)
	Mormon	3 (2.1)
	Other	4 (2.7)
Indigenous community membership	No	138 (94.5)
	Yes	8 (5.5)
Habitual alcohol consumption	No	100 (68.5)
	Yes	46 (31.5)
Alcohol consumption frequency	None	120 (82.2)
	Occasionally	3 (2.1)
	Several times/week (≈3 days/week)	16 (11.0)
	Frequently (4–7 days/week)	7 (4.8)
Smoking (>1 pack/month)	No	129 (88.4)
	Yes	17 (11.6)
Physically active	No	66 (45.2)
	Yes	80 (54.8)
Practices sports regularly	No	87 (59.6)
	Yes	59 (40.4)
History of serious illness	No	111 (76.0)
	Yes	35 (24.0)

Values are presented as mean ± standard deviation (SD) for continuous variables and frequencies (*n*) with percentages (%) for categorical variables. Percentages were calculated using the total study population (*n* = 146). Socioeconomic status was classified according to the Mexican Association of Market Intelligence and Opinion Agencies (AMAI). The AMAI classification includes five socioeconomic levels ranging from E (lowest socioeconomic level), D (low), D+ (lower-middle), C (middle), and C+ (upper-middle), with higher categories representing progressively greater socioeconomic resources and living conditions.

**Table 2:** Comparison of psychological outcomes between the pre-event and post-event assessments.

Outcome	Before (Mean ± SD)	After (Mean ± SD)	Mann–Whitney U	Z	*p*	Effect Size (*r*)
Anxiety-related symptoms (HADS-A)	9.00 ± 3.68	6.12 ± 4.18	6520.0	−5.752	<0.001	0.34
Depressive-related symptoms (HADS-D)	6.41 ± 3.93	6.77 ± 3.68	10,077.5	−0.807	0.420	0.05
Emotional well-being (WHO-5)	48.90 ± 11.80	44.10 ± 11.50	8003.5	−3.703	<0.001	0.22
Sexual attitudes (EROS score)	26.19 ± 3.50	25.25 ± 4.10	9123.5	−2.135	0.033	0.12

Values are presented as mean ± standard deviation (SD). Comparisons between the pre-event and post-event assessments were performed using the Mann–Whitney U test because individual observations could not be paired due to the anonymous study design. Effect size was calculated as *r* = |*Z*|/√*N*, where values of approximately 0.10, 0.30, and 0.50 indicate small, moderate, and large effects, respectively. The EROS assessment-period comparison was exploratory. Given the small effect size and the absence of formal multiplicity adjustment, this finding should be interpreted cautiously. A graphical summary of these multidirectional responses is provided in Supplementary Table S2.

**Table 3:** Beverage consumption during the 8 h preceding the pre-match and post-match assessments.

Beverage	Pre-Match *n* (%)	Post-Match *n* (%)
Water	99 (67.8)	28 (19.2)
Coffee	31 (21.2)	23 (15.8)
Energy drinks	5 (3.4)	8 (5.5)
Juice	6 (4.1)	18 (12.3)
Alcohol	1 (0.7)	60 (41.1)
Flavored water	3 (2.1)	8 (5.5)
Tea	1 (0.7)	1 (0.7)

Values are presented as frequencies (*n*) and percentages (%). Beverage consumption refers to the primary beverage consumed during the eight hours preceding each assessment. The pre-match assessment was conducted on the morning before the Mexico–England FIFA World Cup match, whereas the post-match assessment was conducted on the morning following the match.

**Table 4:** Sex-stratified comparison of psychological outcomes before and after the Mexico–England FIFA World Cup match.

Outcome	Men Pre-Match	Men Post-Match	U	*p*	*r*	Women Pre-Match	Women Post-Match	U	*p*	*r*
Anxiety-related symptoms	7.84 ± 3.21	4.67 ± 3.85	1158.5	<0.001	0.42	9.99 ± 3.78	7.35 ± 4.07	2028.0	<0.001	0.30
Depressive-related symptoms	5.78 ± 3.83	6.22 ± 3.51	2083.0	0.471	0.06	6.95 ± 3.95	7.24 ± 3.77	2997.0	0.666	0.03
Emotional well-being (WHO-5)	47.12 ± 12.32	43.28 ± 11.21	1776.5	0.036	0.18	50.36 ± 11.12	44.88 ± 11.80	2230.5	0.002	0.25
EROS score	26.21 ± 3.48	25.22 ± 3.78	1918.5	0.145	0.13	26.18 ± 3.55	25.27 ± 4.37	2675.0	0.120	0.12

Values are presented as mean ± standard deviation (SD). WHO-5 emotional well-being scores are presented on the standardized 0–100 scale. Comparisons between the pre-match and post-match assessments within each sex were performed using the Mann–Whitney U test. Effect size (*r*) was calculated as *Z*/√*N*, with values of approximately 0.10, 0.30, and 0.50 representing small, moderate, and large effects, respectively.

**Table 5:** Multivariable logistic regression analyses of psychological outcomes at the post-event assessment.

Predictor	Anxiety-Related Symptoms ≥ 8 aOR (95% CI)	*p*	High Well-Being aOR (95% CI)	*p*	Depressive-Related Symptoms ≥ 7 aOR (95% CI)	*p*	Relatively Higher EROS Score aOR (95% CI)	*p*
Female sex	2.78 (1.28–6.04)	0.010	1.45 (0.66–3.19)	0.352	1.13 (0.54–2.40)	0.742	0.94 (0.45–1.94)	0.862
Employment	0.82 (0.39–1.73)	0.606	1.20 (0.55–2.60)	0.651	0.79 (0.38–1.66)	0.539	1.02 (0.50–2.08)	0.962
Smoking history	2.18 (0.74–6.43)	0.159	1.18 (0.37–3.73)	0.775	1.36 (0.45–4.12)	0.586	1.13 (0.39–3.23)	0.824
Regular sports participation	0.70 (0.32–1.53)	0.371	1.57 (0.71–3.45)	0.262	0.23 (0.11–0.49)	<0.001	0.77 (0.37–1.63)	0.501
Recent alcohol consumption (previous 8 h)	0.98 (0.46–2.10)	0.969	1.96 (0.90–4.25)	0.089	1.01 (0.48–2.13)	0.983	0.76 (0.37–1.56)	0.449

All models were based exclusively on post-event observations (*n* = 146). Outcome events were 53 for anxiety-related symptoms, 42 for high emotional well-being, 76 for depressive-related symptoms, and 55 for relatively higher EROS scores. Models were adjusted simultaneously for sex, employment status, smoking history, regular sports participation, and recent alcohol consumption during the eight hours preceding the post-event assessment. Covariates were prespecified and entered simultaneously; no automated or significance-based variable-selection procedure was used. Adjusted odds ratios (aORs) with 95% confidence intervals (CIs) are reported. Anxiety-related symptoms were defined as HADS-A ≥ 8, depressive-related symptoms as HADS-D ≥ 7, and high emotional well-being as WHO-5 > 50. For the exploratory EROS model, the outcome was defined as a score above the sample median (>26), representing a relatively greater erotophilic tendency; scores ≤ 26 represented a relatively greater erotophobic tendency. This median-based categorization was used solely for exploratory analytical purposes and does not represent a validated clinical or diagnostic cutoff. Because these categories are complementary, only the relatively higher EROS category was modeled. Continuous EROS scores were retained for the primary assessment-period comparison.

**Table 6:** Multivariable logistic regression analysis of recent alcohol consumption at the post-event assessment.

Predictor	aOR	95% CI	*p*
Employment	0.29	0.14–0.58	<0.001
Regular sports participation	1.95	0.91–4.19	0.085
Anxiety-related symptoms (HADS-A ≥ 8)	0.94	0.42–2.14	0.890
Depressive-related symptoms (HADS-D ≥ 7)	1.03	0.45–2.38	0.939

The model was based exclusively on post-event observations (*n* = 146), with 60 participants reporting recent alcohol consumption during the eight hours preceding the post-event assessment. Employment status, regular sports participation, anxiety-related symptoms, and depressive-related symptoms were entered simultaneously as prespecified explanatory variables. Adjusted odds ratios (aORs) with 95% confidence intervals (CIs) are reported. Because the outcome and explanatory variables were assessed during the same post-event assessment, associations should be interpreted as cross-sectional and do not establish temporal or causal relationships.

**Table 7:** Sex-stratified multivariable logistic regression analyses of factors associated with recent alcohol consumption during the eight hours preceding the post-event assessment.

Predictor	Men aOR (95% CI)	*p*-Value	Women aOR (95% CI)	*p*-Value
Employment	0.22 (0.08–0.64)	0.005	0.28 (0.10–0.80)	0.017
Regular sports participation	1.07 (0.35–3.26)	0.911	4.13 (1.24–13.71)	0.021
Anxiety-related symptoms (HADS-A ≥ 8)	0.44 (0.11–1.79)	0.253	1.80 (0.56–5.72)	0.321
Depressive-related symptoms (HADS-D ≥ 7)	1.67 (0.49–5.73)	0.415	0.66 (0.19–2.28)	0.513

Abbreviations: aOR, adjusted odds ratio; CI, confidence interval; HADS-A, Hospital Anxiety and Depression Scale–Anxiety subscale; HADS-D, Hospital Anxiety and Depression Scale–Depression subscale. Separate multivariable binary logistic regression models were fitted for men and women using post-event observations only. The dependent variable was self-reported alcohol consumption during the eight hours preceding the post-event assessment (Yes/No). Both models simultaneously included employment status, regular sports participation, anxiety-related symptoms (HADS-A ≥ 8), and depressive-related symptoms (HADS-D ≥ 7) as prespecified explanatory variables. The male model included 67 participants and 30 outcome events, whereas the female model included 79 participants and 30 outcome events. Given the limited number of outcome events relative to the four explanatory variables, these sex-stratified analyses were considered exploratory. Estimates should therefore be interpreted cautiously, particularly those with wide confidence intervals. No formal multiplicity adjustment was applied to these exploratory analyses.

## Data Availability

The original contributions presented in the study are included in the article/Supplementary Material; further inquiries can be directed to the corresponding authors.

## Supplementary Materials

The following supporting information can be downloaded at https://www.mdpi.com/article/10.3390/diseases14090318/s1: Table S1. Reported type of sport according to sex. Table S2. Psychological outcome scores according to assessment period.
