# Supplementary material for: Mental Health and Health-Related Behavioral Patterns Surrounding a Major International Sporting Event: An Observational Study
Source: Diseases. 2026 Aug 31;14(9):318. doi: 10.3390/diseases14090318 (PMC13605560; doi:10.3390/diseases14090318)
Supplement: Supplementary file 1 [file diseases-14-00318-s001.zip › diseases-4500304-supplementary.pdf]

Supplementary material

Table S1. Reported type of sport according to sex.

**Type of sport**	**Male (*n* = 67), n (%)**	**Female (*n* = 79), n (%)**	**Total (*n*= 146), n (%)**
No sport reported	23 (34.33)	46 (58.23)	69 (47.26)
Soccer	11 (16.42)	3 (3.80)	14 (9.59)
Basketball	13 (19.40)	9 (11.39)	22 (15.07)
Volleyball	1 (1.49)	3 (3.80)	4 (2.74)
Swimming	3 (4.48)	1 (1.27)	4 (2.74)
Weightlifting	6 (8.96)	3 (3.80)	9 (6.16)
Athletics	0 (0.00)	2 (2.53)	2 (1.37)
Walking/Jogging	2 (2.99)	1 (1.27)	3 (2.05)
Other	8 (11.94)	11 (13.92)	19 (13.01)
Total	67 (100.00)	79 (100.00)	146 (100.00)

Note: Values are presented as frequency and column percentage, n (%). Percentages were calculated separately for men (***n*** = 67), women (***n*** = 79), and the total sample (***n*** = 146). Each participant was counted once and classified into a single mutually exclusive category according to the type of sport reported. "No sport reported" identifies participants who did not specify a type of sport and should not be interpreted as equivalent to the binary "no regular sports participation" category reported in Table 1. "Other" includes sports not represented by the predefined categories.

Table S2. Psychological outcome scores according to assessment period

Outcome	Pre-event (n = 146), Mean ± SD	Post-event (n = 146), Mean ± SD	Mann–Whitney U	Z	p-value	Effect size (r)
Anxiety-related symptoms (HADS-A)	9.00 ± 3.68	6.12 ± 4.18	6520.0	−5.752	<0.001	0.34
Depressive-related symptoms (HADS-D)	6.41 ± 3.93	6.77 ± 3.68	10077.5	−0.807	0.420	0.05
Emotional well-being (WHO-5; 0–100)	48.90 ± 11.80	44.10 ± 11.50	8003.5	−3.703	<0.001	0.22
Sexual attitudes (EROS score)	26.19 ± 3.50	25.25 ± 4.10	9123.5	−2.135	0.033	0.12

Note: Values are presented as mean ± standard deviation (SD). Each assessment period included 146 observations. Because individual responses could not be linked across assessment periods, pre-event and post-event values represent marginal assessment-period summaries rather than within-participant changes. Comparisons were conducted using the Mann–Whitney U test, and effect sizes were calculated as $r = |Z|/\sqrt{N}$, where N represents the total number of observations included in each comparison. The EROS analysis was exploratory; its nominal *p*-value should be interpreted cautiously because of the small effect size and absence of formal multiplicity adjustment. WHO-5 scores are presented on the standardized 0–100 scale.
